# Griseococcin (1) from *Bovistella radicata (Mont.) Pat* and antifungal activity

**DOI:** 10.1186/s12866-020-01961-x

**Published:** 2020-09-10

**Authors:** Yong Ye, Qinghua Zeng, Qingmei Zeng

**Affiliations:** 1 School of Food and Biological Engineering, Hefei University of Technology, Hefei, 230009 Anhui China; 2 Engineering Research Center of Bio-Process, Ministry of Education, Hefei University of Technology, Hefei, 230009 Anhui China

**Keywords:** Griseococcin, LH-20, DEAE, 1D NMR, 2D NMR, HPLC, FT-IR

## Abstract

**Background:**

To evaluate the antimicrobial and microbicidel activity of *B. radicata* fermentation broth, the broth was purified by DEAE-cellulose and sephadex LC-20 column. The compounds were submitted to spectral analyses (HPLC, FT-IR, 1D and 2D NMR etc.).

**Results:**

The purified compounds were identified as the Griseococcin(s) which were naphthoquinone derivatives, the Chemical formula and MW of Griseococcin (1) was determined as C_37_O_10_H_43_N and 661 Da. only Griseococcin (1) has good antimicrobial activity among the Griseococcin(s). The zone of inhibition (ZOI), minimum inhibitory concentration (MIC) and minimum bactericidal concentration (MBC) or minimum fungicidal concentration (MFC) of Griseococcin (1) were used to investigate the antimicrobial activity. Antifungal activity of Griseococcin (1) was significant, especially for main pathogenic fungus *Trichophyton rubrum* and *Trichophyton mentagrophytes,* MFC/MIC of Griseococcin (1) was 1, while MFC/MIC of postive control was greater than 4, the fungicidal effect of Griseococcin (1) was better than that of positive control.

**Conclusions:**

In this paper, the secondary metabolite compound Griseococcin (1) from *B. radicata* was purified. The purified compound can restrain main pathogens (*T. rubrum* and *T. mentagrophytes*) leading to tinea pedis. The antifungal activity of Griseococcin (1) was similar to that of the positive control and the fungicidal effect of Griseococcin (1) was better than that of positive control, it might be suitable for pharmaceutical industries.

## Background

Tinea pedis is a chronic fungal infection of the feet [1]. Patients that have tinea pedis may be affected by several pathogens, including filamentous fungi named *Trichophyton rubrum* and *Trichophyton mentagrophytes* [2], as well as a yeast named *Candida albicans* [3]. *T. rubrum* is the main pathogenic fungi for tinea pedis, having a prevalence as high as 80% among all tinea-pedis associated pathogenic microbes [4]. Traditionally, to treat tinea pedis, synthetic fungicides such as fluconazole, itraconazole, echinocandins [5], and miconazole nitrate, either by oral medication or external use [6], have been used to treat this disease. Vermes et al. (2000) found that flucytosine and AMB (amphotericin B) were moderately effective in fighting against invasive fungal infections [7–9]. Similar studies on itraconazole have demonstrated that it is effective against fungal infections [10]. However, due to side effects or the continuous drug resistance, some oral medications are unsafe for patients [11], and these chemicals also cause potential deleterious effects on the environment due to their residues [12, 13]. In general, plant natural products have been for decades one of the most successful sources of drugs to treat infectious diseases [14] and natural products extracted represent a rich resource for screening bioactive compounds [15].

Puffballs are widely distributed in many provinces of China, and are various by more than 100 species [16]. *Calvatia gigantean* (*Batsch ex Pers) Lloyd, Calvatia lilacina (Mont.et Berk.) Lloyd, Lasiosphaera fenzlii Reich, Lycoperdon pyriforme Schaeff.:pers*, *Bovistella radicata (Mont.) Pat, Handkea utriformis (HU), H. excipuliformis (HE)*, and *Vascellum pratense (VP)* are all common medicinal puffballs. Although no longer edible in their mature state (because of their powdery consistency), these puffballs have been shown to be a source of active compounds of various biological activities. Puffballs are believed to have several therapeutic properties when used medicinally: hemostasis [17], cough relief [18], suppression of cell division, and antitumor [19] and antimicrobial [20] properties. Petrović P, et al. reported noticeable antimicrobial activity diversity for the methanol extracts obtained from Handkea utriformis (HU), H. *excipuliformis* (HE), and *Vascellum pratense* (VP) [21]. Specimen (*Bovistella radicata (Mont.) Pat*) was dried and deposited in the Institute of Biology, School fo Food and Biological Engineering, Hefei University of Technology (HFUT), China.

The aim of the present study was to evaluate the antimicrobial activity of Griseococcin (1) extracted from *B. radicata* fermentation broth. The antimicrobial and microbicidel activities were evaluated in terms of their minimum inhibitory concentration (MIC), minimum fungicidal concentration (MFC) or minimum bactericidal concentration (MBC) and zone of inhibition (ZOI) values [22], the physico-chemical characterization (HPLC, UV, FT-IR) of Griseococcin (1) and the chemical constituents responsible for this activity were also studied (1D and 2D NMR).

## Results

### Fermentation, extraction and purification of active compound from *B. radicata*

**Fig. 1:**
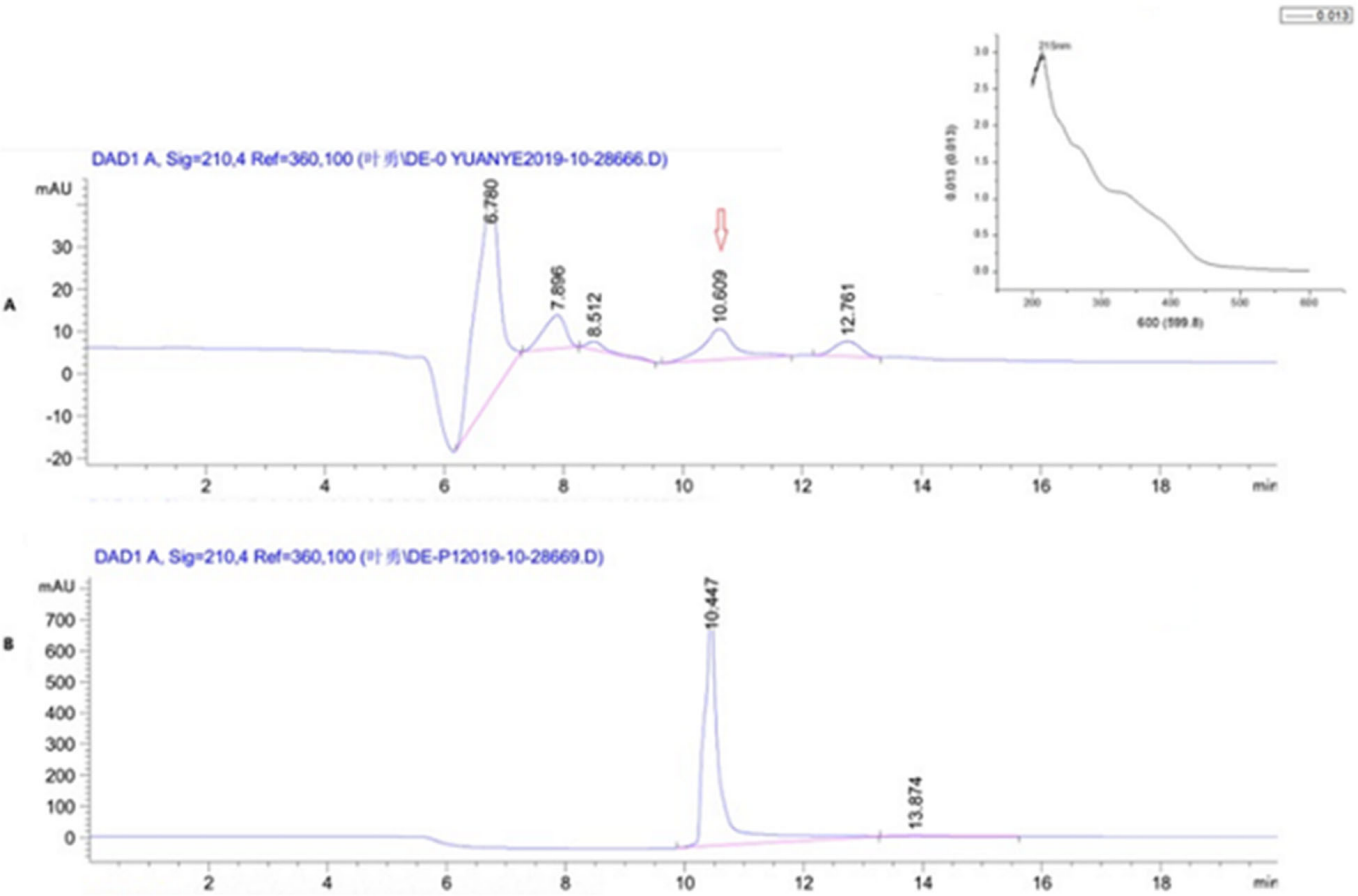
UV spectral and HPLC chromatography of SPAF (**a**) and purified fraction (Griseococcin (1)) (**b**)

20% NaCl elution fraction from fermentation broth of *B. radicata* was named as SPAF by DEAE-cellulose column. The strongest antimicrobial activity fraction from SPAF was Griseococcin (1) by Sephadex LH-20 column. The UV_max_ of all the fraction was 215 nm, the HPLC chromatograms of SPAF and Griseococcin (1) were shown in Fig. 1(a ~ b). The chromatogram of B showed a single and symmetrical peak for Griseococcin (1) (Fig. 1b).

### 1D and 2D NMR of Griseococcin (1)

Griseococcin (1) was isolated as a white amorphous solid powder with the molecular formula of C_37_H_43_NO_10_ derived from the high-resolution electrospray ionization mass spectrum (HR-ESI-MS). The complete assignments for all protons and carbons were shown in Table 1. The ^13^C NMR spectra of Griseococcin (1) displayed signals of thirty seven carbons, including five carbonyl carbons (δC215.7–175.1), five aromatic/olefinic methine carbons (δC 128.86, δC215.7–175.1), seven non-protonated aromatic/olefinic carbons (δC 161.06–109.99), four methyl carbons (δ C20.27)), and four olefin carbons (δC 166.01). The ^1^H NMR spectrum of 1in D_2_O exhibited signals of four methyls at δ H 2.14 (3H, s, H-14′), δ H 2.12 (3H, s, H-15′), δ H 1.06 (3H, s, H-16′) and 1.07 (3H, s, H-17′), five aromatic protons δH 7.80 (1H, s, H-1), δH 7.93(1H, s, H-5), δH 7.72 (1H, s, H-6), δH 7.81 (1H, s, H-8) and 7.66 (1H, s, H-12)], four hydroxyl groups at δ H 8.37 (1H, br s, 4′-OH), δ H 7.81 (1H, br s, 9’OH) and δ H 7.80 (1H, br s, 11′-OH) and 9.63 (1H, br s, 13′-OH).

**Table 1:** Zone of inhibition (ZOI) of Griseococcin (1) on microbial strains

Pathogenic	Zone of inhibition (mm)			
	*T. rubrum*	*T. mentagrophytes*	*E. floccosum*	*C. albicans*
Griseococcin (1)	18.1 ± 0.9	15 .0 ± 1.0	5.0 ± 2.3	1.2 ± 2.2
Terbinafine	20.7 ± 1.6	28.3 ± 2.2	10.02 ± 1.02	6.3 ± 0.6
	*S. aureus*	*E. coli*	*B. subtilis*	*P. aeruginosa*
Griseococcin (1)	10.0 ± 1.9	8.0 ± 1.1	3.0 ± 1.2	3.7 ± 1.4
Gentamicin sulfate	31.7 ± 1.5	28.5 ± 1.4	10.0 ± 1.0	33.6 ± 1.4

**Fig. 2:**
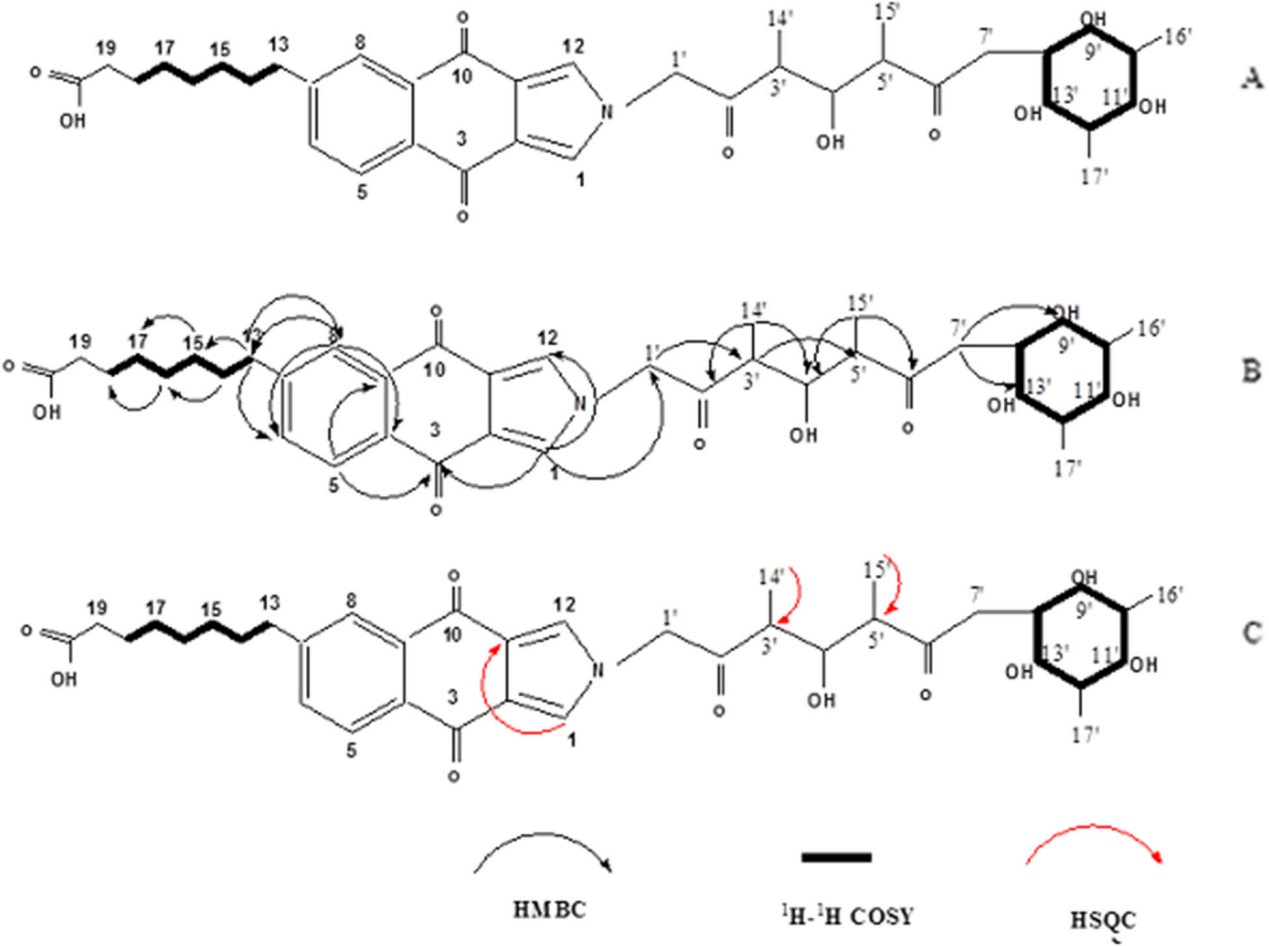
The key ^1^H-^1^H COSY, HMBC and HSQC correlations of Griseococcin (1)

The structure of Griseococcin (1) was deduced by comprehensive analysis of ^1^H-^1^HCOSY, HMBC, and HSQC spectra (Fig. 2a). In Griseococcin (1), the naphthoquinone substructure could be identified by the observation of HMBC correlations from H-8 (δH 7.80) to C-6 (δ C 137.21), C-4 (δC 138.60) and C-13 (δC 30.18), from H-1 (δH 7.81) to C-3 (δC 175.11), C-12 (δC 166.07) and C-1′ (δC 28.40), from H-5 (δH 7.93) to C-3 (δC 175.11) and C-9 (δC 138.56), from H_2_–13 (δH 1.07) to C-8 (δC 135.45) and C-6 (δC 137.21), from H_3_–14′ (δH 1.85) to C-2′ (δC 215.7) and C-4′ (OH) (δC 73.60), from H_3_–15′ (δH 2.11) to C-6′ (δC 215.70) and C-4′ (OH) (δC 73.60), from H_2_–7′ (δH 1.08) to C-9′ (δC 71.25) and C-13′ (δC 71.18). The ^1^H, ^1^H three-bond couplings observed in the COSY spectrum from H-8′ (δH 1.94) to H-9′ (δH 3.62), from H-10′ (δH 1.29) to H-11′ (δH 3.49), from H-12′ (δH 1.73) to H-13′ (δH 3.51), together with the chemical shifts of the ^13^C resonances (C-8′-13′) observed at alternating higher and lower fields, revealed the presence of cyclohexane with alternating hydroxyl and methyl groups. ^1^H-^1^H COSY correlations from H_2_–13 (δH1.07, m) to H_2_–14 (δH3.62, m), from H_2_–14 (δH3.62, m) to H_2_–15 (δH 3.49, m) and from H_2_–16 (δH 3.55, m) to H_2_–17 (δH 3.51, m) and HMBC correlations from H_2_–13 (δH 1,07, m) to C-15 (δC 166.02), from H_2_–14 (δH3.62, m) to C-16 (δC 166), from H_2_–15 (δH3.49, m) to C-17 (δC 166.01) and from H_2_–16 (δH 3.55, m) to C-18 (δC 23.15) identified coupled olefins. The key HMBC correlations from H_2_–1′ (δH1.94, m) to C-3′ (δC 23.4), from H − 3′ (δH2.14, m) to C-5′ (δC 29.05), from H_3_–14′ (δH1.85, m) to C-2′ (δC 215.7) and C-4′-OH (δC73.6), from H3–15′ (δH2.11, m) to C-6′ (δC 215.7) and C-4′-OH identified two meta position carbonyl group and one ortho position hydroxyl group (Fig. 2b).

This connectivity was also secured by the observation of the HSQC correlations from H_3_–14′ to C-3′ and from H_3_–15′ to C-6′. Therefore, the complete structure of naphthoquinone was determined as shown in Fig. 2c.

### Physico-chemical characterization of Griseococcin (1)

Griseococcin (1) was white powder and its solubility was 0.063 g/ml in water. It could be slight soluble in methanol and DMSO, but insoluble in n-hexane, dichloromethane, chloroform, ethyl acetate and acetone.

**Fig. 3:**
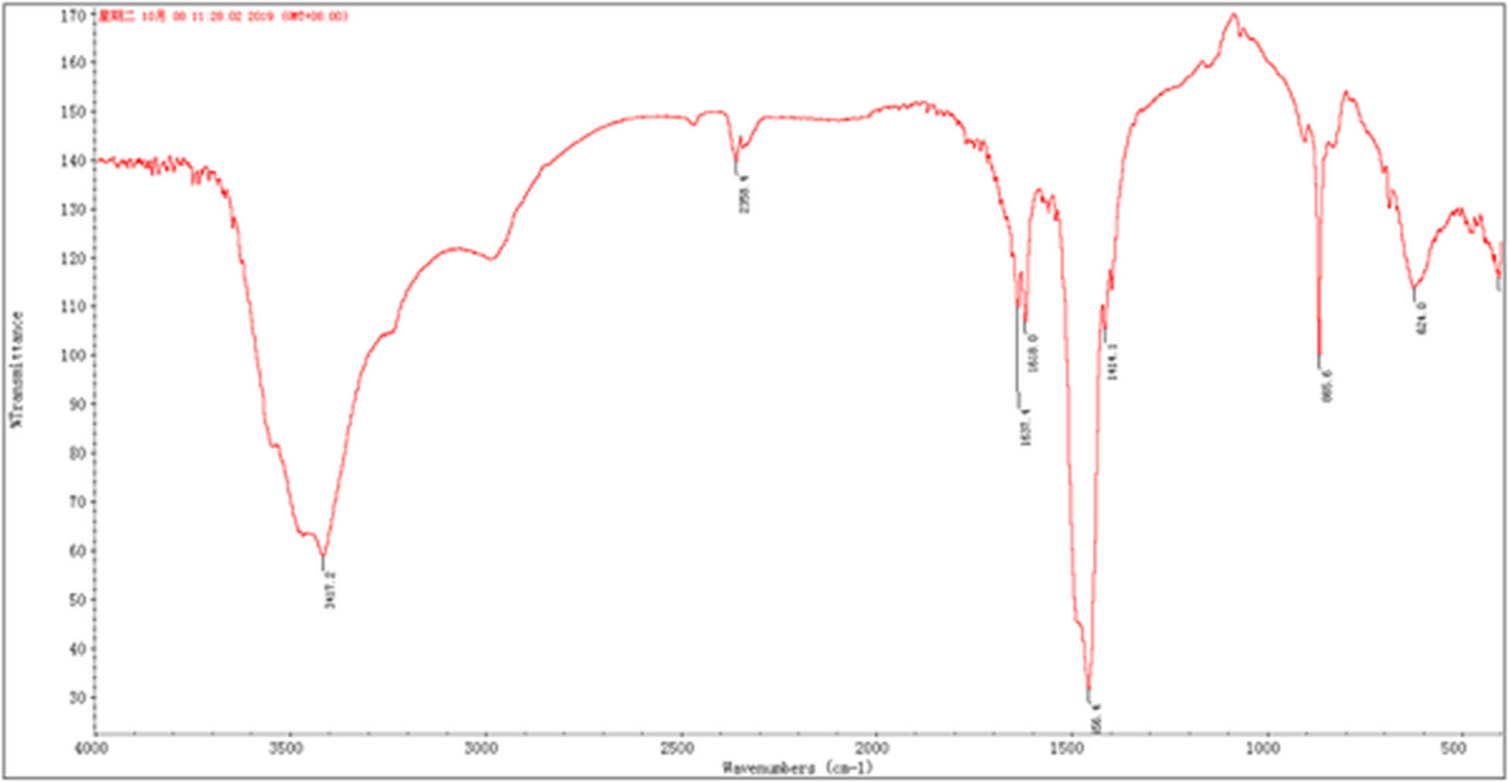
The FT-IR spectrum of Griseococcin (1)

The FT-IR spectrum of Griseococcin (1) showed (Fig. 3) an intense and broad characteristic absorption peaks at 3417.2 cm^− 1^ represented the stretching vibration of O–H. The weak absorption peaks at 2356 and 2925.5 cm^− 1^ were resulted from the stretching vibration of C–H. The absorption bands at 1637.4 and 1618.1 cm^− 1^ are due to the vibration of C=C and C=O in the ester group. The absorptions peaks at 1456.1, 1414 and 624 cm^− 1^ were attributed to the presence of an internal C–H deformation. The strong absorption peak at 866 cm^− 1^ was resulted from aromatics. In conclusion, the typical absorption peak indicated that Griseococcin (1) was naphthoquinone with group O–H,C-H,C=C,C=O and so on [23].

### In vitro antagonistic assay

Griseococcin (1) was assessed for antimicrobial and microbicidel activity against selected *Trichophyton rubrum* (ATCC 28188)*, Trichophyton mentagrophytes* (ATCC 9533)*, Epidermophyton floccosum* (ATCC 52066), *Candida albicans* (ATCC 10231), *Staphylococcus aureus* (ATCC 6538), *Bacillus subtilis* (ATCC 6051), *Escherichia coli* (ATCC 8739) and *Pseudomonas aeruginosa* (ATCC 27582) The results were shown in Table 1, it displayed strong antifungal activity against *T. rubrum, T. mentagrophytes* with ZOI values of 18.06 ± 0.85, 15.01 ± 1.02 mm, as compared to the positive control with ZOI = 20.67 ± 1.58, 28.33 ± 2.15 mm, respectively. While antibacterial activity was weak.

J.Meletiadis et al. reported that compounds were considered bactericidal or fungicidal when the MBC/MIC or MFC/MIC ratio is ≤4 [24]. In this study, it was inportant to discern whether the Griseococcin (1) possesses bactericidal and fungicidal activities. The fungicidal activities of Griseococcin (1) were assessed as MIC, MFC and MFC/MIC. The results were shown in Table 2. Griseococcin (1) showed the high fungicidal activities by means of lowest values of MIC and MFC against the four fungi, especially for main pathogenic fungi (*T. rubrum*), the MIC, MFC and MFC/MIC values were 31.2 ± 2.7, 31.2 ± 3.1 μg/ml and 1, while MIC, MFC and MFC/MIC values of Terbinafine were 15.6 ± 1.2, 31.2 ± 1.6 μg/ml and 6. Fungicidal activities of Griseococcin (1) are revealed more effective than that of commercial reagents (Terbinafine).

**Table 2:** MIC, MFC and MIC/MFC of Griseococcin (1) on fungal strains

Pathogenic fungi	MIC (μg/ml)			
	*T. rubrum*	*T. mentagrophytes*	*E. floccosum*	*C. albicans*
Griseococcin (1)				
MIC (μg/ml)	31.2 ± 2.7	31.2 ± 1.8	250.0 ± 5.2	250 ± 7.6
MFC (μg/ml)	31.2 ± 3.1	31.2 ± 2.1	500 ± 3.1	500 ± 6.7
MFC / MIC	1	1	2	2
Terbinafine				
MIC (μg/ml)	15.6 ± 1.6	7.8 ± 1.2	62.5 ± 2.1	62.5 ± 2.6
MFC (μg/ml)	93.6 ± 2.1	39.0 ± 2.2	250.0 ± 2.1	125 ± 2.6
MFC/ MIC	6	5	4	2

Griseococcin (1) also showed high bactericidal activities with MIC, MBC and MBC/MIC values ranged between 62.5 ~ 125 μg/ml, 125–500 μg/ml and 2–4 against examined bacteria (*S. aureus*, *E. coli* and *P. aeruginosa*). The results were shown in Table 3. Griseococcin (1) showed the highest bactericidal activity for *S. aureus* and *E. coli*,. MBC/MIC value of Griseococcin (1) was 2.0, while the MBC/MIC ratio was 3.0 and 4.0 for positive control (Gentamicin sulfate).

**Table 3:** MIC, MBC and MIC/MFC of Griseococcin (1) on bacterial strains

Pathogenic bacteria	MIC (μg/ml)			
	*S. aureus*	*E. coli*	*B. subtilis*	*P. aeruginosa*
Griseococcin (1)				
MIC (μg/ml)	62.5 ± 1.5	125 ± 2.3	125 ± 1.5	62.5 ± 3.6
MBC (μg/ml)	125 ± 3.1	250 ± 2.1	> 500	250 ± 6.7
MBC / MIC	2.0	2.0	> 4.0	4.0
Gentamicin sulfate				
MIC (μg/ml)	15.6 ± 1.3	31.2 ± 2.4	31.2 ± 3.0	62.5 ± 2.6
MBC (μg/ml)	46.8 ± 1.6	124.8 ± 2.2	202.8 ± 2.1	350 ± 2.2
MBC/MIC	3.0	4 .0	6.5	5.6

Due to side effects and the continuous drug resistance, commercial reagents might be unsafe for patients [11], Therefore, the development of fungicidal therapies is crucial, above results (MIC, MFC or MBC and MFC/MIC or MBC/MIC) add more value to Griseococcin (1).

## Discussion

In the present study, Griseococcin (1) purified from selected puffball (*Bovistella radicata (Mont.) Pat*) had remarkable antifungal activities. These data are consistent with previous findings on the minimum inhibitory concentrations (MICs) and zone of inhibition (ZOI) of *B. radicata* fermentation [20].

According to the Chinese Pharmacopeia, the puffball can restrain *S. aureus* and *P. aeruginosa*. The antifungal function of puffball has not been reported previously, hence, the present study is interesting and original. The novel application of *B. radicata* might be due to different geographic sources of the material used and different strains used [25].

In this study, the purification extraction Griseococcin(s) from fermentation broth of *B. radicata* obtained through celluous DE-52 and sephadex LH-20 column. In Vitro study on antifungal effects of Griseococcin (1) on fungi showed that the most sensitive fungi strains were the main pathogenic fungi (*T. rubrum* and *T. mentagrophytes*) causing tinea pedis, ZOIs were 18.06 ± 0.85 and 15.01 ± 1.02 mm, MICs were 31.2 ± 2.7 and 31.2 ± 1.8 μg/ml, MFCs were 31.2 ± 3.1 μg/ml and 31.2 ± 2.1 μg/ml, MFC/MICs were 1 and 1 against *T. rubrum* and *T. mentagrophytes*. ZOI values of positive control (Terbinafine) were 20.67 ± 1.58 mm 28.33 ± 2.15 mm, MICs were 15.6 ± 1.6 and 7.8 ± 1.2 μg/ml, MFCs were 93.6 ± 2.1 and 39.0 ± 2.2 μg/ml, MFC/MICs were 6 and 5 respectively. The antifungal effect of Griseococcin (1) was similar with that of positive control, the fungicidal effect of Griseococcin (1) was better than that of positive control. The most sensitive bacterial species for Griseococcin (1) was *S. aureus* and *E. coli*, MICs and MBCs were 62.5 ± 1.5, 125 ± 2.3 and 125 ± 3.1, 250 ± 2.1 μg/ml respectively, *P. aeruginosa and B. subtilis* were more resistant. MFC/MICs and MBC/MICs of Griseococcin (1) were less than positive control which meant that antibacterial activity Griseococcin (1) was better than that of the commercial drugs. This study is important for the development of new drugs with low toxicity, overcoming drug resistance and recurrence.

The FT-IR spectrum of Griseococcin (1) showed the strong absorption band, stretching vibration and bending vibration of O-H, C=O, C=C and C-H which belong to a unsaturated coupled bond and aromatic form of naphthoquinone. According to HR-ESI-MS analysis, MW of Griseococcin (1) was 661 Da. Based on the results of different spectral (HPLC, FT-IR, DSC, 1D and 2D NMR etc.) studies and physicochemical properties, the molecular formula of Griseococcin (1) was C_37_H_43_NO_10_ and the molecular structure of Griseococcin (1) was shown in Fig. 1. MBC/MICs of were 6 and 5 respectively.

Previously, many authors reported the various biological activity of fermentation broth from puffball like anticancer activity [26, 27], antioxidant activity [28], antifatigue effect [29], etc. In the present study, the antifungal activity of *B. radicata* was another important biological function. The biological activities of organic compounds are related to their molecular weight, functional groups, the length of chain, the composition of group and the number of branches, hydrophilic and hydrophobic group. It means that the structure-activity relationship should be disclosed.

## Conclusions

Future work concentrating on determining the antifungal mechanisms of Griseococcin (1) will be performed, which will be helpful in laying a foundation for overcoming the drug resistance that pathogens quickly develop against tinea pedis.

In this paper, the antifungal secondary metabolite compound Griseococcin (1) from *B. radicata* were studied. The compound from *Bovistella radicata (Mont.) Pat* was purified. Molecular weight and molecular formula of the purified compound (Griseococcin (1)) were 661 Da and C_37_H_43_NO_10_ respectively, it can restrain main pathogens (*T. rubrum* and *T. mentagrophytes*) leading to tinea pedis. The antifungal activity of Griseococcin (1) was similar to that of the positive control.

## Methods

### Sample *Bovistella radicata (Mont.) Pat* collection and tested microorganisms

*Bovistella radicata (Mont.) Pat* was obtained from Shuinan town, Jishui county, Jiangxi province, China. After strain identification and authenticated by Professor Qingmei Zeng, it belongs to *Agaricalesorder*, *Lycoperdaceae family*, *Bovistella genus*, *Bovistella radicata (Mont.) Pat* species. The tested pathogenic fungi included *Trichophyton rubrum* (ATCC 28188)*, Trichophyton mentagrophytes* (ATCC 9533)*, Epidermophyton floccosum* (ATCC 52066), and *Candida albicans* (ATCC 10231)*.* Four strains of test pathogenic bacteria included *Staphylococcus aureus* (ATCC 6538), *Bacillus subtilis* (ATCC 6051), *Escherichia coli* (ATCC 8739) and *Pseudomonas aeruginosa* (ATCC 27582). All standard bacterial and fungal strains were obtained from rom the Microbiology Laboratory at Department of Biology, Anhui Medical University, Anhui.

### Fermentation, extraction and purification of *Bovistella radicata (Mont.) Pat*

The mature *B.radicata* should be dried at 40 °C for at least 2 days, and taken out when its weight is no longer changed, the sporophore and spore powder were ground together and filtered through a 100 mesh sieve. The mixed powder of *B.radicata* was inoculated into 100 mL of potato dextrose broth (PDB) in 250 ml flask. The flask was kept in rotary shaker at 25 °C with 115 rpm for 72 h. The pH and moisture content of PDB was also determined according Maguireboyle (2014) and Mcauliffe (2016) [30, 31]. For every 12 h, the fermentation was taken to perform antimicrobial activity against main pathogens *T. rubrum* and *T. mentagrophytes* by zone of inhibition (ZOI) method. Then the fermentation were centrifuged at 7000 rpm for 20 min and filtered over Whatman No.4 paper to get the final clear supernatant and preserve at 4 °C. 50 ml clear supernatant was purified firstly using 100 ml DEAE-cellulose column and eluted by different concentration NaCl (10–30%) to get different fractions. 20% NaCl elution fraction showed best antifungal activity against pathogens and was named as SPAF. Furthermore, SPAF (20% NaCl elution fraction) was purified by sephadex LH-20 column. Different purified fractions (named Griseococcin(s)) were obtained from SPAF, only Griseococcin(1) (500 μg/ml) has antifungal activity and it’s biochemical characteristics and spectral (HPLC, FT-IR, 1D and 2D NMR etc.) studies were assessed.

### Antimicrobial activity

The examined methods were the minimum inhibitory concentrations (MICs) [22], minimum bactericidal concentration (MBCs) or minimum fungicidal concentration (MFCs) [32, 33] and zone of inhibitions (ZOIs) [34]. ZOI is qualitative analysis and MIC is quantitative analysis of antimicrobial activity [35]. The MICs, MBCs and MFCs of Griseococcin (1) were determined in the 96-well plates by the double micro dilution method against pathogens. 100 μL dilutions (approximately 10^6^ CFU/mL) of *T. rubrum, T. mentagrophytes, E. floccosum,* and *C. albicans* in potato dextrose broth and *S. aureus*, *B. subtilis, E. coli* and *P. aeruginosa* in Nutrient Broth [36] were inoculated into 96-well plates. Then, 100 μL Griseococcin (1) solutions were added after a double dilution with the corresponding medium broth (from 500 μg/mL to7.8 μg/mL). 0.9% (v/v) NaCl was used as the negative control. Gentamicin sulfate or Terbinafine were dissolved in normal saline (NS) to a concentration of 1 mg/mL for the subsequent tests as positive control against bacteria or fungi. The Petri dishes were incubated at 37 °C for 24 h with *S. aureus*, *E. coli, B. subtilis* and *P. aeruginosa*, for 48 h with *T. rubrum, T. mentagrophytes, E. floccosum,* and *C. albicans*. Griseococcin (1) was also dissolved in NS at 1 mg/mL. The MIC was recorded as the lowest concentration of sample showing no detectable growth. MFC or MBC was determined as the concentration causing no visible growth and killing 99.5% of the original inoculum. Ten microliters of sub-inhibitory concentrations of Griseococcin (1) was placed in the corresponding solid medium for 48 h to determine the MBC or MFC values according to the growth of the microbial colonies. Each sample was performed twice. The zones of inhibition (ZOI) of Griseococcin (1) (100 μg/ml) was also evaluated. The prepared Griseococcin (1) was filled into the wells. After incubating for 24 h at 37 °C, the measurements were done basically from the edge of the zone to the edge of the well [34].

### General experimental procedures

The UV_max_ absorption spectrum of SPAF was analyzed at full-wave spectra (200–900 nm) by UV/vis 2802 spectrophotometer. The FT-IR spectrum of Griseococcin(s) were recorded on a Thermo Nicolet Spectrum FT-IR in a range of 4000–400 cm^− 1^ with KBr pellets. HR-ESI-MS data were obtained on an Agilent 1260 Infinity LC coupled to a 6230 TOF. 20 mg of the dried sample was dissolved in 0.55 mL of deuteroxide (99.99% D) in a NMR tube. 1D and 2D NMR spectra were acquired on an AVANCE-600 NMR spectrometer (Bruker Inc., Rheinstetten, Germany) at 50 °C. The chemical shifts were given in δ (ppm) and referenced to the solvent signal (D_2_O-d_6_, δ H 2.50, δ C 39.5). Column chromatography (CC) was conducted on DEAE-cellulose and Sephadex LH-20. The fractions Griseococcin(s) were also monitored by HPLC (Agilent 1260 chromatography system, USA) which was equipped with a diode array detector (DAD). The DAD detector was set at 215 nm to acquire chromatograms. The separation of the compound was performed on a Hypersil RP-C18 column (5 μm, 250× 10.0 mm, Thermo Fisher Scientific, USA) at a temperature of 25 °C. Injection volume: 20 μL.

### Griseococcin (1)

Griseococcin (1): IR (neat) v max 3417, 2926, 2356, 1637, 1618, 1456, 1414, 866, 624 cm^− 1^; UV (D_2_O) λ max 215 nm; ^1^H and ^13^CNMR data see Table 4; HR-ESI-MS m/z 661.1970 [M + H] + (calcd for C_37_ H_43_NO_10_, 661.1968).

**Table 4:** ^1^H (700 MHz) and ^13^CNMR (175 MHz) spectroscopic data for Griseococcin (1): in DMSO

Position	δ H (mult, J inHz)	δC
1	7.81	166
2		128.56
3		175.11
4		138.60
5	7.93	135.7
6	7.72	137.21
7		136.28
8	7.80	135.45
9		138.56
10		175.07
11		128.57
12	7.66	166.07
13	1.07	30.18
14	3.62	166.01
15	3.49	166.02
16	3.55	166.03
17	3.51	166.01
18	1.25	23.15
19	1.94	23.16
20	2.09	184.15
1′	1.94	28.4
2′		215.7
3′	2.14	23.4
4′	4.47	73.6
4′-OH	8.37	
5′	2.12	29.05
6′		215.7
7′	1.08	25.52
8′	1.94	26.81
9′	3.62	71.25
9′-OH	7.81	
10′	1.29	23.45
11′	3.49	71.15
11′-OH	7.80	
12′	1.73	23.47
13′	3.51	71.18
13′-OH	7.93	
14′	1.85	20.27 CH_3_
15′	2.11	20.27 CH_3_
16′	1.06	20.27 CH_3_
17′	1.07	20.27 CH_3_

## Supplementary information

**Additional file 1.**

## Data Availability

The datasets used and analysed during the current study are available from the corresponding author on resonable request.

## Abbreviations

NMR: Nuclear Magnetic Resonance
HPLC: High Performance Liquid Chromatography
FT-IR: Fourier transform infrared spectroscopy
DMSO: Dimethyl Sulfoxide
ZOI: Zone of inhibition
MIC: Minimum inhibitory concentration
HR-ESI-MS: High-resolution electrospray ionization mass spectroscopy
DAD: Diode array detector
MW: Molecular weight
