# Supplementary material for: Griseococcin (1) from Bovistella radicata (Mont.) Pat and antifungal activity
Source: BMC Microbiol. 2020 Sep 10;20:276. doi: 10.1186/s12866-020-01961-x (PMC7488052; doi:10.1186/s12866-020-01961-x)
Supplement: Supplementary file 1 — Additional file 1. [file 12866_2020_1961_MOESM1_ESM.docx]

**A point-by-point response letter**

**Point by point answers for editor:**

1. Please ensure the author names on the title page match that on the system - currently the first and last names are reversed.

2. In the "Availability of Data and Materials" section, please detail where the raw data supporting your findings can be found (including information found in any supplementary files). If the raw data is publicly available or can be requested, please state that this is the case, and where the data can be found/requested from. Alternatively, if you do not wish to/cannot share your data, please state (in the ‘Availability of data and Materials’ section) that data will not be shared, and state the reason.

3. Please note the order of initials when referring to an author in the Authors' contributions should be [first initial of first name][first initial of middle name, if applicable][first initial of last name]. Please amend the order of initials in regard to each author as appropriate.

For authors with the same intials (QZ). To distinguish between them in the Authors’ Contributions section please designate them as QZ1 and QZ2, with QZ1 corresponding to Qinghua Zeng, chosen because this name is furthest up the author list.

4. Please remove the point by point responses/ manuscript with tracked changes from the manuscript.

5. At this stage, please upload your manuscript as a single, final, clean version that does not contain any tracked changes, comments, highlights, strikethroughs or text in different colours. All relevant tables/figures/additional files should also be clean versions. Figures (and additional files) should remain uploaded as separate files. Please ensure that all figures, tables and additional/supplementary files are cited within the text.

**Answers:**

1. I have changed corresponding author qingmei zeng on the system to Qingmei Zeng which consistent with the name in manuscript.
2. In the "Availability of Data and Materials" section, the datasets used and analysed during the current study are available from the corresponding author on resonable request.
3. In the Authors' contributions, Qinghua Zeng was designated as QZ1 and Qingmei Zeng was designated as QZ2.
4. The point by point manuscript with tracked changes was removed.
5. The uploaded manuscript and all relevant tables/figures/additional files are clean version. All figures, tables and additional/ supplementary files could be cited in the text. All figures were uploaded as separate files.

**Point by point answers for reviewer:**

Reviewer 2:

1) Words are often stuck together (please add spaces).

**Answer:** Corrected

e.g. Background section, line 1-2, 5,11-12,14, 19, page3, et al;

Result section, line 30, page 4; line 14,31, page 5, et al;

Discussion section, line 8-15, page7, et al;

Methods section, line12-15, line 22-23, page 8; line1-5, page 9, et al.
